# Thrombospondin-1 Restricts Interleukin-36γ-Mediated Neutrophilic Inflammation during Pseudomonas aeruginosa Pulmonary Infection

**DOI:** 10.1128/mBio.03336-20

**Published:** 2021-04-06

**Authors:** Hernán F. Peñaloza, Tolani F. Olonisakin, William G. Bain, Yanyan Qu, Rick van der Geest, Jill Zupetic, Mei Hulver, Zeyu Xiong, Michael W. Newstead, Chunbin Zou, Jonathan K. Alder, Joel A. Ybe, Theodore J. Standiford, Janet S. Lee

**Affiliations:** a Acute Lung Injury Center of Excellence, Division of Pulmonary, Allergy, and Critical Care Medicine, Department of Medicine, University of Pittsburgh, Pittsburgh, Pennsylvania, USA; b Pulmonary and Critical Care Medicine, Department of Medicine, University of Michigan, Ann Arbor, Michigan, USA; c Department of Environmental and Occupational Health, School of Public Health, Indiana University, Bloomington, Indiana, USA; University of Alabama at Birmingham; University of Mississippi Medical Center

**Keywords:** *Pseudomonas aeruginosa*, thrombospondin-1, IL-36γ, proteolytic environment

## Abstract

Interleukin-36γ (IL-36γ), a member of the IL-1 cytokine superfamily, amplifies lung inflammation and impairs host defense during acute pulmonary Pseudomonas aeruginosa infection. To be fully active, IL-36γ is cleaved at its N-terminal region by proteases such as neutrophil elastase (NE) and cathepsin S (CatS). However, it remains unclear whether limiting extracellular proteolysis restrains the inflammatory cascade triggered by IL-36γ during P. aeruginosa infection. Thrombospondin-1 (TSP-1) is a matricellular protein with inhibitory activity against NE and the pathogen-secreted *Pseudomonas* elastase LasB—both proteases implicated in amplifying inflammation. We hypothesized that TSP-1 tempers the inflammatory response during lung P. aeruginosa infection by inhibiting the proteolytic environment required for IL-36γ activation. Compared to wild-type (WT) mice, TSP-1-deficient (Thbs1^−/−^) mice exhibited a hyperinflammatory response in the lungs during P. aeruginosa infection, with increased cytokine production and an unrestrained extracellular proteolytic environment characterized by higher free NE and LasB, but not CatS activity. LasB cleaved IL-36γ proximally to M^19^ at a cleavage site distinct from those generated by NE and CatS, which cleave IL-36γ proximally to Y^16^ and S^18^, respectively. N-terminal truncation experiments *in silico* predicted that the M^19^ and the S^18^ isoforms bind the IL-36R complex almost identically. IL-36γ neutralization ameliorated the hyperinflammatory response and improved lung immunity in Thbs1^−/−^ mice during P. aeruginosa infection. Moreover, administration of cleaved IL-36γ induced cytokine production and neutrophil recruitment and activation that was accentuated in Thbs1^−/−^ mice lungs. Collectively, our data show that TSP-1 regulates lung neutrophilic inflammation and facilitates host defense by restraining the extracellular proteolytic environment required for IL-36γ activation.

## Abstract (precis)

Pseudomonas aeruginosa pulmonary infection can lead to exaggerated neutrophilic inflammation and tissue destruction, yet host factors that regulate the neutrophilic response is not fully known. IL-36γ is a proinflammatory cytokine that dramatically increases in bioactivity following N-terminal processing by proteases.

## INTRODUCTION

Pneumonia is a major cause of global mortality in children and older adults (1–3) and is the most common risk factor of acute respiratory distress syndrome (ARDS) in the intensive care unit (ICU) (4). Pseudomonas aeruginosa, a Gram-negative bacterium that commonly causes acute lower respiratory tract infection in the ICU, is associated with prolonged mechanical ventilation and increased morbidity and mortality during ARDS (5, 6). P. aeruginosa is also a major cause of chronic lung infection in cystic fibrosis patients, and the emergence and dissemination of extensively drug-resistant or multidrug-resistant P. aeruginosa isolates poses an increasing risk to human health (7, 8). Epithelial cells and resident alveolar macrophages within the lower respiratory tract and alveolar space sense P. aeruginosa, releasing a multitude of cytokines (e.g., interleukin 6 [IL-6], IL-1β, IL-8, and tumor necrosis factor alpha [TNF-α]) and chemokines (CXCL-1 and CXCL-2) (9, 10) that promote the recruitment and activation of neutrophils. Robust neutrophilic inflammatory response, while essential for P. aeruginosa clearance (9, 10), requires rapid curtailment in order to limit bystander tissue damage (11).

Thrombospondin-1 (TSP-1) is a matricellular glycoprotein (12–14) whose function is defined contextually by binding to structural matrix proteins (laminins, fibronectin, and collagen), cell surface molecules such as proteoglycans, receptors, or integrins, and other soluble mediators such as cytokines and proteases (13, 15–19). Originally identified as a secreted protein involved in stabilizing a provisional fibrin clot at sites of injury (20–26), our prior work has shown that TSP-1 dampens an excessive inflammatory response and regulates extracellular protease function in the lung (27–29). How inflammation and extracellular proteases are linked mechanistically in the context of TSP-1 remains unresolved. We previously reported that TSP-1 harbors a Kazal-like consensus sequence shared by some serine protease inhibitors in its type 3 repeat domain that restrains the activity of neutrophil serine proteases *in vivo* (28). We also showed that TSP-1 is cleaved by the pathogen-encoded protease LasB into two fragments but retains its inhibitory action against LasB and NE to limit neutrophilic inflammation during severe P. aeruginosa respiratory infection (27).

The IL-36 family is comprised of three different proinflammatory cytokines (IL-36α, IL-36β, and IL-36γ) and one anti-inflammatory cytokine (IL-36Ra), which are part of the IL-1 superfamily of cytokines (that includes IL-1α, IL-1β, IL-18, IL-33, and IL-38). IL-36 cytokines, and in particular IL-36γ, exert a critical role in host immunity during acute respiratory infections (30, 31). IL-36 cytokines bind to a common IL-36 receptor (IL-1Rrp2), triggering the recruitment of the IL1RAcP accessory protein and leading to signal transduction through MyD88 and downstream MAPK and NF-κB activation (32, 33). IL-36γ appears to be protective during lung infection caused by different pathogenic bacteria such as Streptococcus pneumoniae, Klebsiella pneumoniae, and Legionella pneumophila (30, 34). In contrast to these infections, IL-36γ is paradoxically harmful following P. aeruginosa infection-induced injury, as IL-36R-deficient (IL-36R^−/−^) and IL-36γ^−/−^ mice are protected from excessive host inflammatory response and show improved lung bacterial clearance (31). These findings suggest that while IL-36γ is important in early host defense, excessive inflammation mediated by IL-36γ may be a rational target against P. aeruginosa-induced lung tissue damage.

Several studies have identified the proteases involved in IL-36γ processing and activation. Cathepsin S cleaves IL-36γ just proximal to S^18^ (35), and this product has been identified as the most active form of IL-36γ *in vitro* (33, 35). Other studies, however, have shown that neutrophil serine proteases such as neutrophil elastase (NE) and proteinase-3 cleave IL-36γ proximal to Y^16^, resulting in an active form that can amplify the inflammatory response (36–38). In this study, we show that the pathogen-derived protease LasB, in addition to host-derived protease NE, can cleave IL-36γ and that the absence of TSP-1 dramatically amplifies the inflammatory and neutrophil response triggered by IL-36γ during P. aeruginosa infection.

## RESULTS

### Thrombospondin-1 limits excessive proinflammatory cytokine production and neutrophil-dominant immune cell recruitment during acute P. aeruginosa intrapulmonary infection.

**FIG 1:**
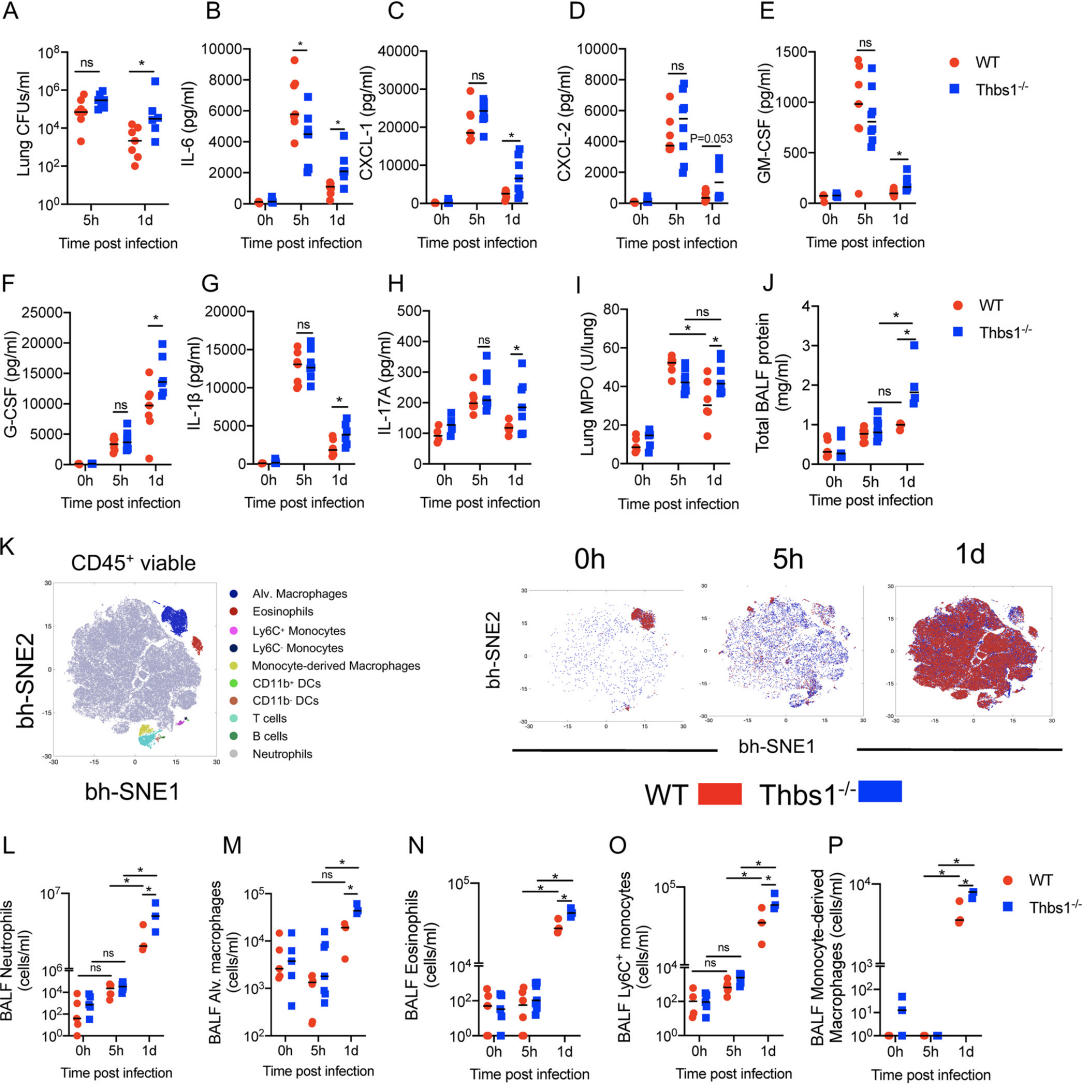
Thrombospondin-1 (TSP-1) prevents excessive inflammatory cell recruitment by restraining the production of cytokines and chemokines in the lungs during P. aeruginosa infection. TSP-1-deficient (Thbs1^−/−^) and WT mice were intratracheally (i.t.) inoculated with P. aeruginosa at an inoculum of 10^6^ CFU. (A) Lung bacterial burden (CFU/ml) was measured at 5 h postinfection (hpi) and at 1 day postinfection (dpi). In parallel, (B) IL-6, (C) CXCL-1, (D) CXCL-2, (E) GM-CSF, (F) G-CSF, (G) IL-1β, (H) IL-17A, and (I) myeloperoxidase (MPO) activity were measured in lung tissue homogenates after 5 hpi and 1 dpi. (J) Total bronchoalveolar lavage fluid (BALF) protein content was measured after 5 hpi and 1 dpi. (K) Immunophenotyping of BALF leukocytes was analyzed by the unbiased Barnes-Hut modification of t-SNE (bh-SNE) method using live CD45^+^ cells from WT and Thbs1^−/−^ mouse samples at 0 h, 5 h, and 1 day postinfection. (Left) Clusters of leukocyte subsets based upon expression level of surface markers. (Right) Kinetics of leukocyte subsets in BALF of WT and Thbs1^−/−^ mice at 0 h, 5 h, and 1 day postinfection. Quantification of gated (L) neutrophils, (M) alveolar macrophages, (N) eosinophils, (O) Ly6C^+^ monocytes, and (P) monocyte-derived macrophages from WT and Thbs1^−/−^ mice at 5 hpi and 1 dpi. *, *P < *0.05 for single comparisons; the Shapiro-Wilk test was used to assess normal distribution followed by a Mann-Whitney U test or a parametric *t* test. A two-way analysis of variance (ANOVA) test was followed by a *post hoc* test for multiple comparisons over time. Each data point represents an individual mouse, combined from two independent experiments. Lines indicate the median.

**FIG 2:**
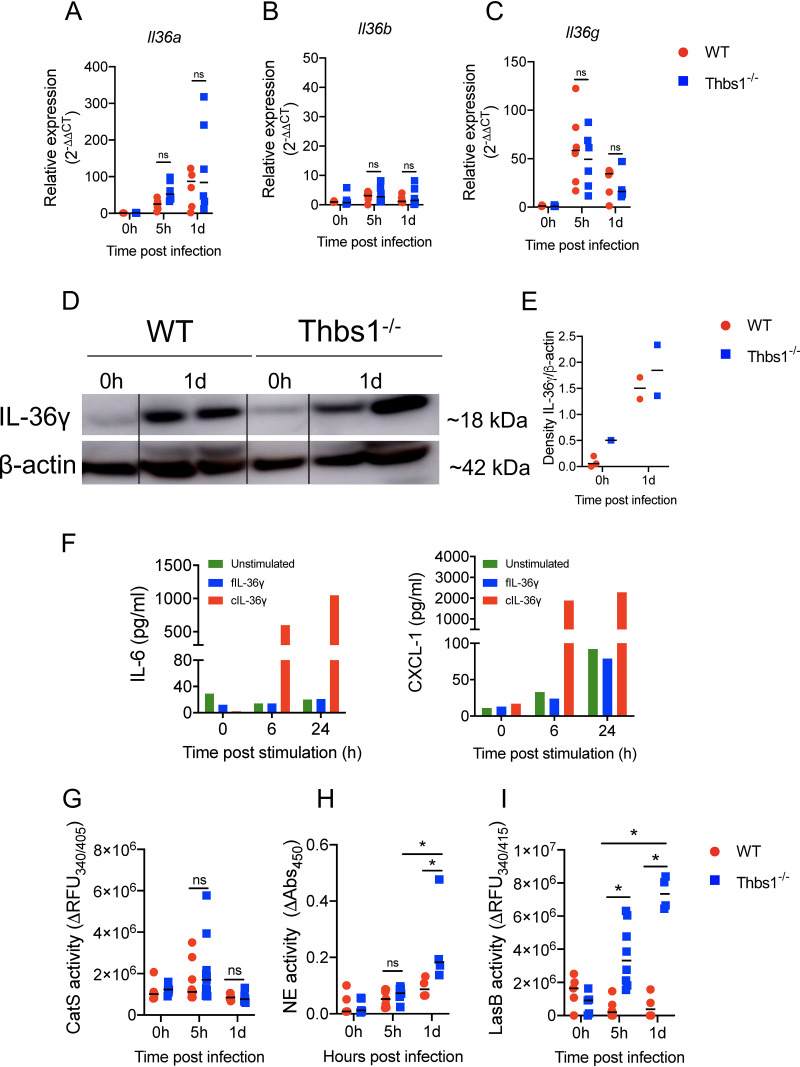
TSP-1 does not alter IL-36 cytokines expression but downregulates the proteolytic environment required for activation. Thbs1^−/−^ and WT mice were i.t. inoculated with P. aeruginosa at an inoculum of 10^6^ CFU, and lung tissue (A) *Il36a*, (B) *Il36b*, and (C) *Il36g* transcripts were measured at 5 hpi and 1 dpi by quantitative reverse transcription-PCR (qRT-PCR) using *gadph* as the internal housekeeping gene. (D and E) IL-36γ expression in the lungs measured by Western blot at 1 dpi. Density expression of IL-36γ is normalized to β-actin. (F) IL-6 and CXCL-1 production by bone marrow-derived dendritic cells (BMDCs) after stimulation with full-length (fIL-36γ) or cleaved IL-36γ (cIL-36γ, S^18^ isoform). (G) Cathepsin S (CatS), (H) neutrophil elastase (NE), and (I) LasB activity were measured in the BALF of WT and Thbs1^−/−^ mice at 5 hpi and 1 dpi using the specific substrates 2-aminobenzoyl-l-alanyl-glycyl-l-leucyl-l-alanyl-*para*-nitro-benzyl-amide, *N*-methoxysuccinyl-Ala-Ala-Pro-Val *p*-nitroanilide, and Mca-GRWPPMG∼LPWEK(Dnp)-*D*-R-NH2, respectively. *, *P < *0.05, for single comparisons, the Shapiro-Wilk test was used to assess normal distribution, followed by a Mann-Whitney U test or a parametric *t* test. A two-way ANOVA test was followed by a *post hoc* test for multiple comparisons over time. Each data point represents an individual mouse, combined from two independent experiments, except for the Western blot and *in vitro* BMDC stimulation, which were performed once. Lines indicate the median.

We previously reported increased lung bacterial burden and exaggerated neutrophilic inflammation in *Thbs1^−/−^* mice at 20 h postinfection with P. aeruginosa compared to wild-type (WT) mice (27). To better understand the mechanism underlying the early inflammatory response, we examined the kinetics of infection at 5 h postinfection (hpi), when no differences in bacterial burden were detected, and at 1 day postinfection (dpi), where the absence of TSP-1 resulted in increased bacterial burden in the lungs (Fig. 1A). At 5 hpi, both WT and *Thbs1^−/−^* mice experienced a rapid increase in several proinflammatory cytokines, such as IL-6, CXCL-1, CXCL-2, granulocyte-macrophage colony-stimulating factor (GM-CSF), granulocyte colony-stimulating factor (G-CSF), IL-1β, and IL-17A (Fig. 1B to H). Although both Thbs1^−/−^ and WT mice produced similar amounts of most cytokines analyzed at 5 hpi, WT mice produced higher levels of IL-6 at this time postinfection (Fig. 1B), indicating early differences in the immune response of these mice against P. aeruginosa. At 1 dpi, however, Thbs1^−/−^ mice showed increased levels of IL-6, CXCL-1, CXCL-2, GM-CSF, G-CSF, IL-1β, and IL-17A in the lungs at 1 dpi compared to WT mice (Fig. 1B to H). We observed sustained myeloperoxidase (MPO) content in the lungs at 5 hpi and 1 dpi in Thbs1^−/−^ mice, in contrast to WT mice, where lung MPO peaked at 5 hpi but was downregulated by 1 dpi (Fig. 1I). Furthermore, Thbs1^−/−^ mice showed increased lung microvascular permeability, as evidenced by increased total bronchoalveolar lavage fluid (BALF) protein compared to that in WT mice (Fig. 1J). The early production of cytokines and chemokines induced a robust recruitment of leukocytes to the BALF of WT and Thbs1^−/−^ mice. Flow cytometry analyses enabled determination of immune cell infiltration into the airspaces of WT and Thbs1^−/−^ mice, where neutrophil numbers showed a mild and nonsignificant increase at 5 hpi compared with 0 hpi but were significantly elevated by 1 dpi (Fig. 1K; see also Fig. 1A and 2 and Table S1 in the supplemental material). Notably, other cells, such as resident alveolar macrophages, eosinophils, classical (Ly6C^+^) and alternative (Ly6C^−^) monocytes, dendritic cells (DCs), B cells, T cells, and monocyte-derived macrophages, were found in the BALF of both WT and Thbs1^−/−^ mice (Fig. 1K). However, Thbs1^−/−^ mice exhibited increased numbers of neutrophils, alveolar macrophages, eosinophils, Ly6C^+^ monocytes, and monocyte-derived macrophages (Fig. 1L to P). Thbs1^−/−^ mice also showed elevated numbers of T cells and B cells but equivalent numbers of CD11b^+^ DCs, CD11b^−^ DCs, and Ly6C^−^ monocytes (see Fig. S3 in the supplemental material). These data suggest that TSP-1 restrains proinflammatory response by 1 dpi in the lungs and limits excessive recruitment of neutrophils and other myeloid cells into the airspaces, enhancing P. aeruginosa clearance and reducing lung injury.

10.1128/mBio.03336-20.3 FIG S3 T cells, B cells, dendritic cells, and Ly6C^−^ monocytes in the BALF of WT and Thbs1^−/−^ mice in response to PA14 infection. WT and Thbs1^−/−^ mice were intratracheally (i.t.) inoculated with 10^6^ CFU of PA14. (A) T cells, (B) B cells, (C) CD11b^+^ DCs, (D) CD11b^neg^ DCs, and (E) Ly6C^−^ monocytes were identified and quantified by flow cytometry at 5 hpi and 1 dpi. A two-way analysis of variance (ANOVA) test was followed by a *post hoc* test for multiple comparisons over time. Each data point represents an individual mouse and two independent experiments. Lines indicate the median. Download FIG S3, PDF file, 0.08 MB. Copyright © 2021 Peñaloza et al. 2021 Peñaloza et al. https://creativecommons.org/licenses/by/4.0/ This content is distributed under the terms of the Creative Commons Attribution 4.0 International license.

10.1128/mBio.03336-20.5 TABLE S1 Surface phenotypes of recruited leukocytes in BALF during P. aeruginosa infection. Download Table S1, PDF file, 0.02 MB. Copyright © 2021 Peñaloza et al. 2021 Peñaloza et al. https://creativecommons.org/licenses/by/4.0/ This content is distributed under the terms of the Creative Commons Attribution 4.0 International license.

### Thrombospondin-1 does not alter IL-36γ expression induced by P. aeruginosa infection but restrains the proteolytic activity of NE and LasB that can mediate IL-36γ cleavage at distinct sites.

IL-36 cytokines are major effectors of the immune response in the lungs during P. aeruginosa and other bacterial infections (30, 31, 34). *Il36a* transcript level was increased in the lungs by 1 dpi (Fig. 2A), but no changes in expression were detected for *Il36b* (Fig. 2B). *Il36g* transcript level peaked at 5 hpi but remained increased above baseline at 1 dpi (Fig. 2C). However, there were no differences in *Il36a*, *Il36b*, and *Il36g* transcriptional responses between WT and Thbs1^−/−^ mice. In addition, we noted increased levels of IL-36γ protein in the lungs at 1 dpi in both WT and Thbs1^−/−^ mice (Fig. 2D and E). As N-terminal processing of IL-36γ is required for full bioactivity and the triggering of proinflammatory cytokines IL-6 and CXCL-1 by IL-36R in bone marrow-derived dendritic cells (BMDCs) and human keratinocytes (39, 40), we show that cleaved IL-36γ (cIL-36γ) just proximal to S^18^ but not full-length IL-36γ (fIL-36γ) leads to a robust production of IL-6 and CXCL-1 by BMDCs *in vitro* (Fig. 2F). The protease responsible for IL-36γ cleavage in keratinocytes is cathepsin S (CatS), which cleaves IL-36γ into the potent S^18^ isoform (35). However, we were unable to identify substantial differences in BALF CatS activity in WT and Thbs1^−/−^ mice following P. aeruginosa infection (Fig. 2G), prompting us to hypothesize that other proteases may be involved in the N-terminal processing and activation of IL-36γ in the lungs. Notably, Thbs1^−/−^ mice showed higher levels of BALF free NE activity at 1 dpi (Fig. 2H), and higher BALF P. aeruginosa LasB activity at 5 hpi and 1 dpi (Fig. 2I). Although TSP-1 does not regulate IL-36 cytokine gene and protein expression, these data show that TSP-1 tempers the proteolytic environment of the lung, which is potentially required to boost the biological activity of IL-36γ protein.

**FIG 3:**
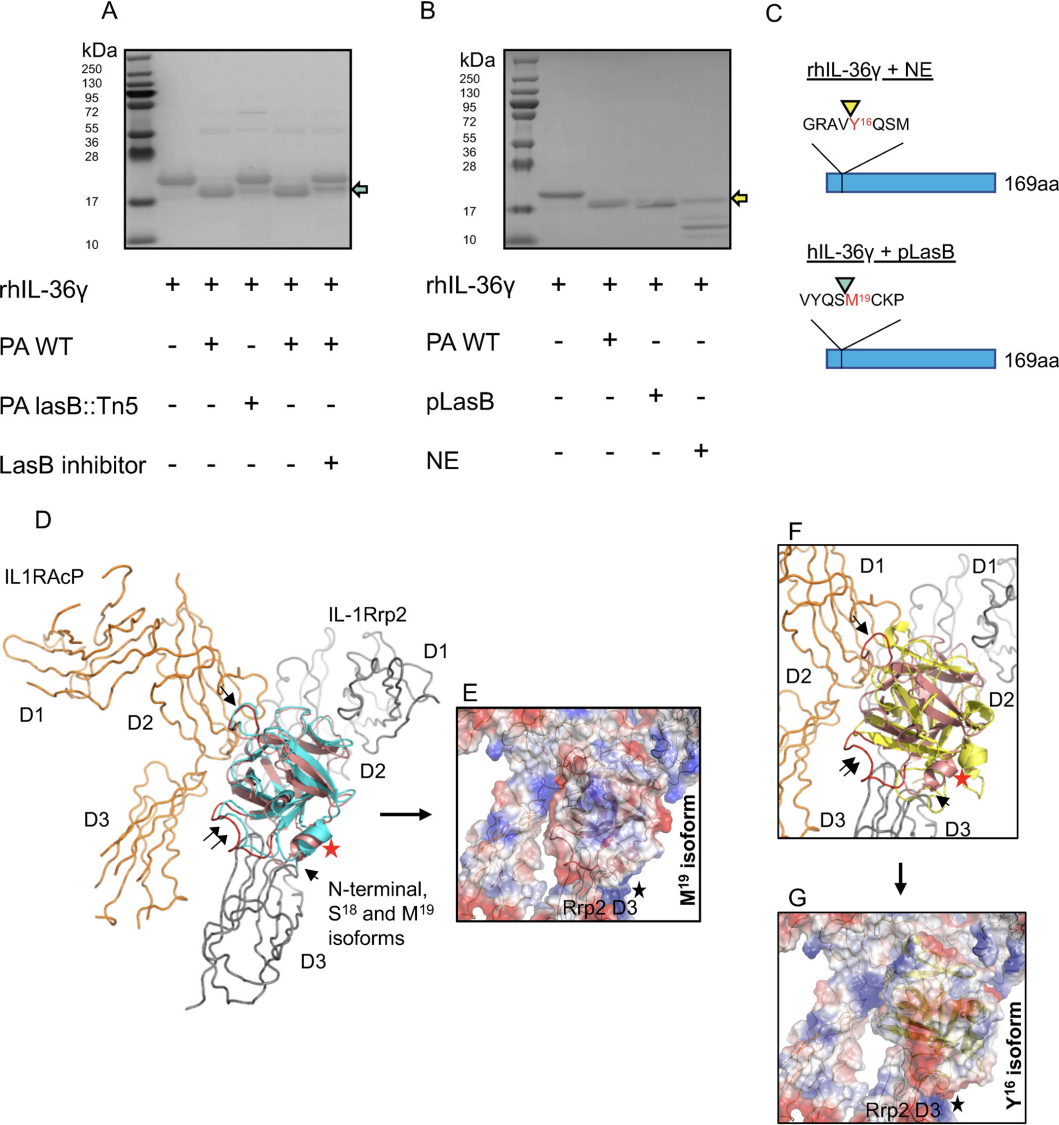
PA14 LasB cleaves IL-36γ proximally to M^19^, and sequential N-terminal truncation models *in silico* predict the bioactivity of the M^19^ isoform. (A) Full-length IL-36γ was incubated with wild-type PA14 (PA WT) or *lasB*::Tn*5* mutant supernatant in the presence or absence of LasB inhibitor. (B) Full-length IL-36γ was incubated with PA WT, purified LasB (pLasB), or recombinant NE and visualized by SDS-PAGE. Teal arrow points to M^19^ IL-36γ, and yellow arrows point to Y^16^ IL-36γ. (C) N-terminal sequencing of IL-36γ NE and IL-36γ LasB were analyzed by Edman degradation, with arrowheads indicating the site of cleavage. (D) IL-36γ S^18^ (salmon) and M^19^ (teal) isoforms associate in a similar orientation to a model of the IL-1Rrp2/IL1RAcP receptor complex (gray and orange, respectively). Parts of D2 and D3 of IL-1Rrp2 and the D2 domain of IL1RAcP contribute to the identified cytokine-binding site. Helix I^104^-G^109^ (red star) is close to the N termini of both isoforms pointed toward the IL-1Rrp2 D3 domain. The single black arrow indicates loop L^155^-N^160^ (red), which makes favorable electrostatic contact with the IL1RAcP D2 domain. Loop T^61^-D^72^ (double black arrow, red loop) faces the D3 domain of the accessory protein. (E) The electrostatic pattern of M^19^ isoform binding may influence the IL-1Rrp2 D3 domain. The black star denotes a predicted concentration of electrostatic repulsions (basic charge in blue) exist at the lower contact interface between the isoform and the IL-1Rrp2 D3 domain. (F) Y^16^ isoform (yellow) binds in the upside-down fashion, but there are differences compared to the M^19^ isoform (see position shift of the landmark helix [red star]). For reference, the single and double black arrows again indicate the loops shown in in panel D. (G) The Y^16^ isoform binds in the upside-down arrangement, but the intermolecular packing is less efficient between the cytokine and IL-36R complex. The figures were prepared and electrostatic potential surfaces calculated using PyMol Molecular Graphics System v1.3 (Schrodinger, LLC).

The major neutrophil protease responsible for IL-36γ activation *in vitro* is NE (37), and NE has been reported to cleave IL-36γ proximally to Y^16^ and Q^17^ (here referred to as Y^16^ and Q^17^ isoforms) (35), although only the Y^16^ isoform has been previously shown to be biologically active (35, 37). We evaluated whether LasB, a pathogen-derived metalloprotease with elastase activity, can cleave IL-36γ. Incubation of human full-length IL-36γ (18.7 kDa) with cell-free supernatant of P. aeruginosa grown in culture resulted in the cleavage of IL-36γ to a smaller product of approximately 17 kDa (Fig. 3A). Supernatant obtained from a transposon insertion mutant of P. aeruginosa strain PA14 deficient in LasB (PA14*lasB*::Tn*5*) (27) or PA14 WT in the presence of a LasB inhibitor reduced the cleavage of IL-36γ (Fig. 3A). These findings suggest that the PA-protease LasB can cleave IL-36γ. We next compared the cleavage of IL-36γ by purified LasB (pLasB) and NE. Our data show that LasB and NE cleave IL-36γ at different positions of the N-terminal region (Fig. 3B). N-terminal sequencing was conducted by Edman degradation and showed that LasB cleaved IL-36γ just proximally to M^19^ (M^19^ isoform), whereas NE-mediated cleavage of IL-36γ yielded several products with the largest truncated product at Y^16^ (Fig. 3C). The latter finding is consistent with previous reports (35–38).

### Sequential N-terminal truncation models *in silico* predict the bioactivity of the M^19^ isoform.

The N-terminal truncation of IL-36γ just proximal to S^18^ (S^18^ isoform) amplifies the bioactivity of IL-36γ by ∼1,000-fold *in vitro* (33, 35). To gain molecular insight into why the bioactivity of IL-36γ is dependent on the removal of N-terminal residues, we set out to evaluate *in silico* how a panel of IL-36γ sequential truncation models could bind to the IL-1Rrp2/IL-1RAcP heterodimer (IL-36R) complex. To avoid any steric clashes with IL1RAcP during docking runs, we used a previously reported homology model of the IL-36R complex (41). Since the N terminus before S^18^ was unresolved in the IL-36γ crystal structure (PDB identifier 4IZE), we employed the deep learning algorithm RaptorX (http://raptorx.uchicago.edu) to compute a model of the full-length IL-36γ (fIL-36γ) molecule for truncation, as the three-dimensional (3D) structure of the S^18^-D^169^ amino acid sequence of the model generated by RaptorX was a close match to the 4IZE crystal structure with the same sequence. The binding patterns of our collection of N-terminal deletion models were determined in a series of docking trials using ClusPro 2.0. In the first round of docking, we tested the −3, −6, −9, −12, −15, −18, and −21 amino acid IL-36γ truncation models. The fIL-36γ model (as well as the −3, −6, −9, and −12 models) did not bind in a way deemed productive based on the work of others (41, 42). After the coarse sampling, we proceeded to analyze the docking between IL-36R with the Y^16^, S^18^, and M^19^ isoforms, produced by NE, CatS, and LasB, respectively.

The M^19^ isoform [ClusPro 2.0 job identifier [ID] 456743: cluster 0 (95 members), −1099 weighted lowest energy score] sits in a groove composed of parts from the IL-1Rrp2 D2 and D3 domains. Importantly, the N terminus, next to a short helix (I^104^-G^109^, indicated with a red star), faces the receptor D3 domain (Fig. 3D). In this arrangement, the bound cytokine is upside down relative to the orientation of the IL-36R complex. Moreover, loop L^155^-N^160^ is nestled in a shallow pocket in the D2 domain of IL-1RAcP, while loop T^61^-D^72^ faces the D3 domain of the accessory protein (Fig. 3D). Interestingly, our upside-down orientation bears a strong resemblance to an earlier prediction of how the 4IZE crystal structure binds to the receptor complex (42). Last, there is a network of favorable electrostatic interactions between the M^19^ isoform and the IL-36R complex (Fig. 3E). Our binding model also contains a set of destabilizing electrostatic repulsions (Fig. 3E, black star), potentially suggesting the existence of a complex interplay between different electrostatic forces in guiding the docking of proteolytically cleaved IL-36γ.

The repose of the M^19^ isoform is almost identical to how the S^18^ isoform binds (Fig. 3D) [job ID 456742: cluster 5 (46 members), −892 weighted lowest energy score]. Thus, we conclude that the S^18^ and M^19^ isoforms may interact with IL-1Rrp2 in a close, if not identical, fashion, offering a structural rationale for why the pLasB cleavage product, M^19^ isoform, might possess strong bioactivity. Finally, we asked how the Y^16^ isoform could associate with the IL-36R complex. Y^16^ isoform [job ID 456740: cluster 3 (43 members), −883 weighted lowest energy score] can adopt the basic upside-down binding orientation, but is out of sync by a counterclockwise rotation compared to the position of M^19^ isoform helix (I^104^-G^109^) (Fig. 3F). As for packing, there are fewer atomic contacts between the Y^16^ isoform and IL-1RAcP in the IL-36R complex, suggesting the Y^16^ isoform has a looser upside-down fit. Our docking-modeling data suggest that electrostatic interactions may contribute to the docking stability of the Y^16^ isoform. Unlike S^18^ and M^19^ isoforms, we do not see any electrostatic repulsions that could influence any potential movement of the IL-1Rrp2 D3 domain (Fig. 3G, black star). This raises the possibility that favorable electrostatic contacts may counteract the looser fit of IL-36γ to its receptor complex. This suggestion could prove to have implications for the strength of the IL-36γ-mediated inflammatory response.

### Neutralization of IL-36γ improves lung immunity and inflammatory response against P. aeruginosa in the absence of thrombospondin-1.

**FIG 4:**
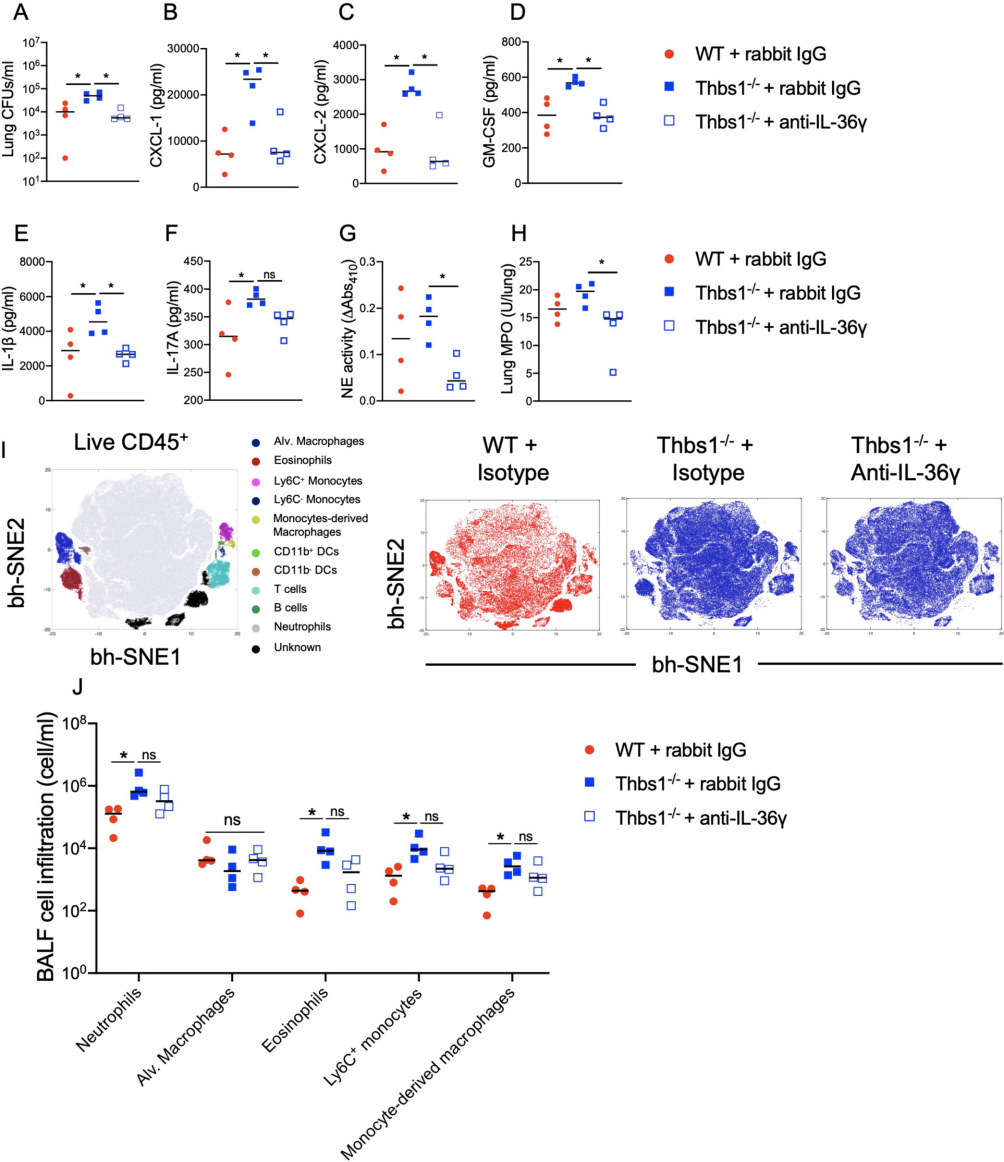
IL-36γ neutralization reduces lung bacterial burden and the inflammatory response during P. aeruginosa infection in Thbs1^−/−^ mice. Thbs1^−/−^ and WT mice were i.t. inoculated with P. aeruginosa at an inoculum of 10^6^ CFU. After 5 hpi, Thbs1^−/−^ mice were treated with a rabbit anti-mouse IL-36γ neutralizing antibody. Thbs1^−/−^ and WT mice treated with a rabbit-IgG served as control. At 1 dpi, (A) lung bacterial burden, (B) CXCL-1, (C) CXCL-2, (D) GM-CSF, (E) IL-1β, and (F) IL-17A production in lung tissue homogenates, (G) BALF free NE activity, (H) lung tissue MPO activity, and (I and J) immune cell composition in the BALF were measured. *, *P < *0.05 by one-way ANOVA test followed by a *post hoc* test. Each data point represents an individual mouse; the experiment was performed once without excluding any data. Lines indicate the median.

To evaluate whether the hyperinflammatory response observed in Thbs1^−/−^ mice at 1 dpi is mediated by IL-36γ, we intraperitoneally (i.p.) injected Thbs1^−/−^ mice with a neutralizing rabbit anti-mouse IL-36γ antibody or rabbit IgG (30) at the time point of 5 h during the peak of *Il36g* expression. IL-36γ neutralization at 5 hpi had a major protective effect in Thbs1^−/−^ mice, as evidenced by improved P. aeruginosa burden in the lungs (Fig. 4A), a significant reduction of proinflammatory cytokines and chemokines such as CXCL-1, CXCL-2, GM-CSF, and IL-1β (Fig. 4B to E), and a nonsignificant reduction of IL-17A, G-CSF, or IL-6 (Fig. 4F; see also Fig. S4A and B in the supplemental material). Moreover, IL-36γ neutralization reduced free BALF NE (Fig. 4G) and lung tissue MPO (Fig. 4H) activity. Although the infiltration of neutrophils and other immune cells such as eosinophils, macrophages, Ly6C^+^ monocytes (Fig. 4I and J; see also Fig. S1B in the supplemental material), DCs, Ly6C^−^ monocytes, and T and B cells (Fig. S4C) were not significantly reduced in the BALF after IL-36γ neutralization, these data show that IL-36γ mediates the hyperinflammatory response in the lungs of Thbs1^−/−^ mice during P. aeruginosa infection and supports the hypothesis that TSP-1 tempers IL-36γ activity.

10.1128/mBio.03336-20.1 FIG S1 Unbiased expression pattern of different cell markers used to identify immune cell subsets by Barnes-Hut modification of t-SNE (bh-SNE). Viable CD45^+^ cells of (A) bronchoalveolar lavage fluid (BALF) from wild-type WT and thrombospondin-1-deficient (Thbs1^−/−^) mice at 5 hpi and 1 dpi after P. aeruginosa lung infection. (B) WT and Thbs1^−/−^ mice treated with an anti-interleukin 36γ (IL-36γ) or control IgG analyzed at 1 day postinfection (dpi). (A and B) Bar indicates relative fluorescence expression level of surface markers in viable leukocyte subsets of a composite BALF sample. Red indicates high-level expression population; blue indicates low-level expression population. Download FIG S1, PDF file, 2.4 MB. Copyright © 2021 Peñaloza et al. 2021 Peñaloza et al. https://creativecommons.org/licenses/by/4.0/ This content is distributed under the terms of the Creative Commons Attribution 4.0 International license.

10.1128/mBio.03336-20.4 FIG S4 Cytokine production and infiltration pattern of dendritic cells, T cells, B cells, and Ly6C^−^ monocytes in the BALF of PA14-infected WT and Thbs1^−/−^ mice following IL-36γ neutralization. Thbs1^−/−^ and WT mice were i.t. inoculated with P. aeruginosa at an inoculum of 10^6^ CFUs. After 5 hpi, Thbs1^−/−^ mice were treated with a rabbit anti-mouse IL-36γ neutralizing antibody. Thbs1^−/−^ and WT mice treated with a rabbit-IgG antibody served as control arms. At 1 dpi, (A) G-CSF and (B) IL-6 production were measured in the lung tissue homogenates. In parallel, (C) CD11b^+^ DCs, CD11b^−^ DCs, T cells, B cells, and Ly6C^−^ monocytes were identified and quantified by flow cytometry in the BALF. Each data point represents an individual mouse. Lines indicate the median. Download FIG S4, PDF file, 0.07 MB. Copyright © 2021 Peñaloza et al. 2021 Peñaloza et al. https://creativecommons.org/licenses/by/4.0/ This content is distributed under the terms of the Creative Commons Attribution 4.0 International license.

### Thrombospondin-1 regulates neutrophil function and proinflammatory cytokine production induced by N-terminally processed IL-36γ in the lungs.

**FIG 5:**
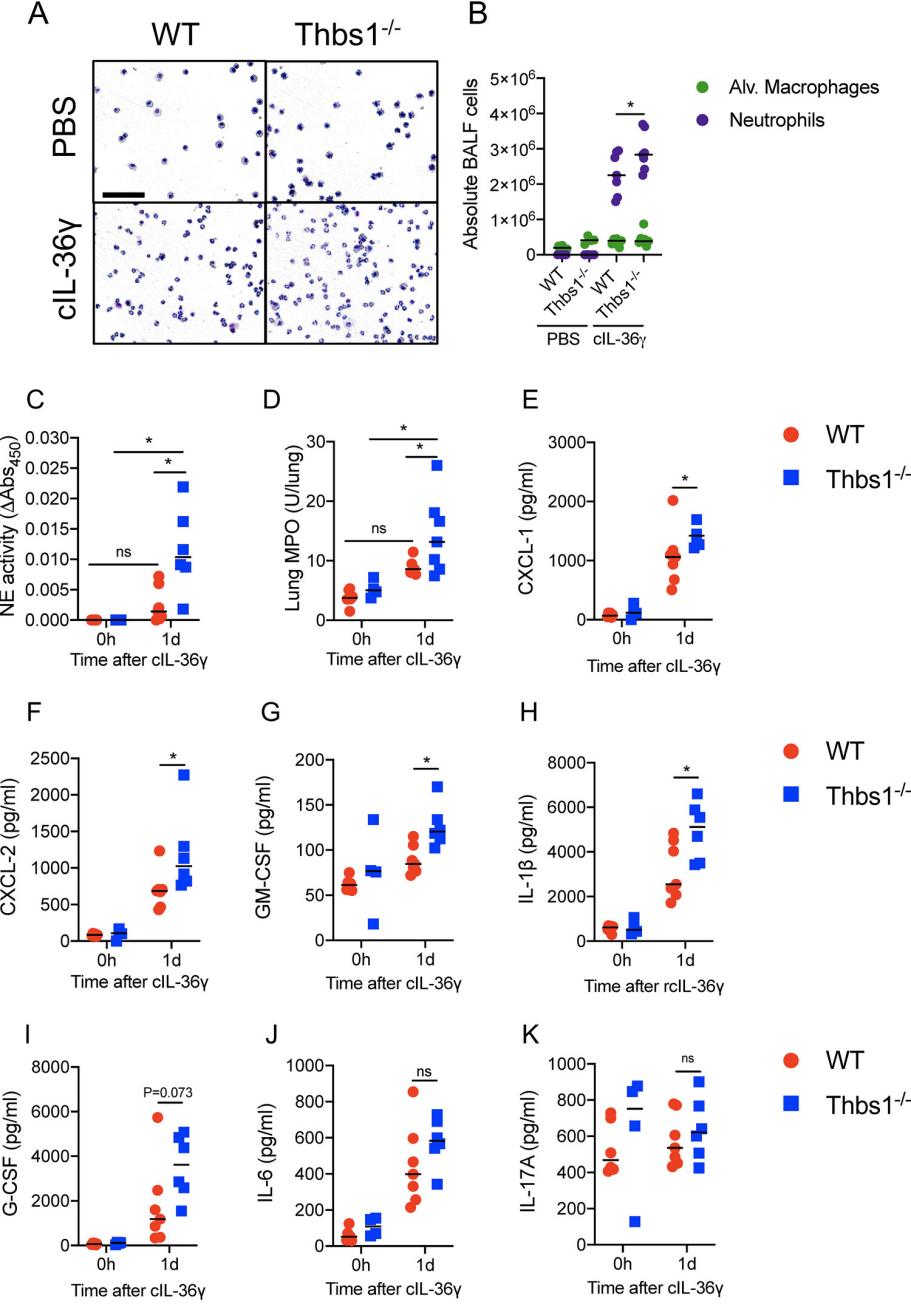
TSP-1 tempers neutrophil recruitment and activation and lung cytokine production induced by cleaved IL-36γ. Thbs1^−/−^ and WT mice were i.t. instilled with 2.5 μg of cleaved IL-36γ (S^18^ isoform). At 1 dpi, (A and B) BALF cytospin results showing airspace neutrophil recruitment and BALF macrophages and neutrophils were measured by flow cytometry. (C) BALF free NE and (D) lung tissue MPO were measured in the BALF. In parallel, (E) CXCL-1, (F) CXCL-2, (G) GM-CSF, (H) IL-1β, (I) G-CSF, (J) IL-6, and (K) IL-17A were measured in the lungs. Bar, 100 μm. *, *P < *0.05 for single comparisons; the Shapiro-Wilk test was used to assess normal distribution, followed by a Mann-Whitney U test or a parametric *t* test. A two-way ANOVA test was followed by a *post hoc* test for multiple comparisons over time. Each data point represents an individual mouse and two independent experiments. Lines indicate the median.

We next evaluated whether TSP-1 directly regulates the inflammatory effects triggered by IL-36γ in the lungs. As cleaved IL-36γ, but not full-length IL-36γ, induced the production of IL-6 and CXCL-1 in murine BMDCs (Fig. 2F), we intratracheally delivered cIL-36γ (S^18^ isoform) to WT and Thbs1^−/−^ mice. One day posttreatment, we evaluated the influx and activation of neutrophils, as well as cytokine and chemokine production. Instillation of cIL-36γ induced robust neutrophil recruitment to the airspaces, as measured in the BALF of WT and Thbs1^−/−^ mice (Fig. 5A). However, Thbs1^−/−^ mice showed higher airspace neutrophil counts (Fig. 5B) and elevated free NE activity (Fig. 5C) and lung tissue MPO activity (Fig. 5D) compared to that in WT mice. In addition, Thbs1^−/−^ mice showed exaggerated proinflammatory chemokine and cytokine response in the lungs, including CXCL-1, CXCL-2, GM-CSF, and IL-1β (Fig. 5E to H). G-CSF and IL-6 (Fig. 5I and J) were induced by cIL-36γ but were not significantly different between WT and Thbs1^−/−^ mice, whereas IL-17A was not induced by cIL-36γ (Fig. 5K). The findings indicate that, in the absence of TSP-1, neutrophil recruitment, activation, and inflammatory cytokine production induced by cIL-36γ are amplified in the lungs.

## DISCUSSION

Our findings indicate TSP-1 regulates the inflammatory response in the lungs mediated by IL-36γ during P. aeruginosa lower respiratory tract infection by restraining the extracellular proteolytic environment. Compared to WT mice, Thbs1^−/−^ mice developed a hyperinflammatory response in the lungs during P. aeruginosa infection that is characterized by enhanced production of proinflammatory cytokines and chemokines, as well as by increased influx of neutrophils and other leukocytes. While IL-36γ is induced in the lungs early during infection, WT and Thbs1^−/−^ mice showed similar levels of IL-36γ in both transcript and protein expression. Others have shown that the bioactivity of IL-36 cytokines requires N-terminal processing by proteases such as NE *in vitro* (37), and here we show that cleaved IL-36γ (S^18^ isoform), but not full-length IL-36γ, induced IL-6 and CXCL-1 production in BMDCs. Instillation of cIL-36γ recapitulated the amplified proinflammatory cytokine and chemokine response and enhanced neutrophil influx and activation observed with P. aeruginosa infection in Thbs1^−/−^ mice. Moreover, IL-36γ neutralization reduced the production of proinflammatory cytokines and free neutrophil NE activity in the lungs of Thbs1^−/−^ mice and paradoxically improved the ability of Thbs1^−/−^ mice to clear P. aeruginosa in the lungs. Together, our data provide evidence that TSP-1 tempers the hyperinflammatory response during P. aeruginosa lung infection by regulating IL-36γ bioactivity and restraining feed-forward inflammation.

Once full-length IL-36γ is secreted to the extracellular space, host proteases cleave the protein and thereby increase its bioactivity ∼500- to 1,000-fold, allowing IL-36γ to bind to the IL-36R complex and trigger inflammation (33, 35–37). NE, proteinase-3, and CatS are host proteases that cleave and activate IL-36γ *in vitro* (33, 35–37). However, little is known regarding pathogen-derived proteases that can directly cleave the IL-36 family of cytokines and about the regulation of extracellular proteases *in vivo*. Given the notable increase in free NE and LasB activity in the lungs of Thbs1^−/−^ mice, we examined N-terminal processing of IL-36γ by NE and LasB and show that NE and LasB cleave IL-36γ just proximally to Y^16^ and M^19^, respectively. Sequential truncation experiments performed *in silico* predict that the M^19^ isoform and bioactive S^18^ isoform show a similar binding pattern to that of the IL-36R complex, a heterodimer formed by IL-1Rrp2 and IL1RAcP. Mechanistically, the binding of IL-36γ to IL-1Rrp2 prompts the recruitment of IL1RAcP. One study has proposed that the binding of IL-36γ can drive the intermolecular association of the D3 domains of the receptor and accessory protein (41). Consequently, their Toll/interleukin-1 receptor (TIR) domains (tethered to D3s), along with the TIR domain of the adaptor protein MyD88, form a signaling platform comprised of an intermolecular TIR domain trimer. This leads to the activation of NF-κB, which will traffic to the nucleus to modulate the transcription of a set of genes, including those that encode proinflammatory cytokines (41). Although our docking models are static snapshots, the concentration of like charges in close physical proximity raises the possibility that electrostatic interactions may play a role in D3 domain association. In our model, the activity of CatS—the protease responsible to process IL-36γ into the S^18^ isoform (35)—was not significantly increased in the airspaces following P. aeruginosa infection. CatS contributes to several processes in the extracellular space, including degradation of the extracellular matrix (43, 44). However, CatS also participates in the intracellular antigen processing required for MHC-I and MHC-II class antigen presentation (45). Therefore, it is possible that CatS may be involved in the intracellular processing of full-length IL-36γ, although this remains to be seen.

Mice deficient in TSP-1 show exaggerated neutrophilic response to cleaved IL-36γ, which suggests the existence of a feed-forward mechanism in which neutrophils that arrive into the airspaces release more proteases to further amplify inflammation. Following intratracheal administration, cleaved IL-36γ leads to enhanced production of chemokines and cytokines, presumably by resident IL-36R^+^ cells in the lungs (31, 46, 47). We suggest that recruited neutrophils release proteases into the airspaces that cleave and activate *de novo* synthesized IL-36γ that further drives neutrophil recruitment. We further suggest that TSP-1 limits this feed-forward inflammation mediated by IL-36γ by restraining the extracellular proteolytic environment in the lung during P. aeruginosa infection. What might trigger the initial N-terminal processing of IL-36γ during infection? It is conceivable that a pathogen-secreted protease could serve this role. Indeed, our findings show that a pathogen protease, LasB, can cleave IL-36γ to generate the M^19^ isoform, which is predicted to have a similar binding pattern to IL-36R as the bioactive S^18^ isoform of IL-36γ. Together, these data indicate that the restraint of host and pathogen proteases that cleave full-length IL-36γ into different isoforms is a key checkpoint that regulates the biological activity of IL-36γ. In this context, our data suggest that, during P. aeruginosa infection, the inhibitory properties of TSP-1 over NE and LasB control the generation of IL-36γ isoforms implicated in runaway neutrophilic inflammation.

P. aeruginosa-infected patients with ARDS show increased BALF and plasma levels of IL-36γ (31). ARDS is a heterogeneous syndrome, and at least two endotypes of ARDS have been identified, a hyperinflammatory and a hypoinflammatory endotype (48). The hyperinflammatory endotype is characterized by a robust production of IL-8, IL-6, and TNF-α and is associated with a high mortality (48, 49). These three cytokines are related to IL-36γ either as downstream or upstream effectors (33, 46, 50), suggesting that IL-36γ could be a potential contributor to the development of the hyperinflammatory endotype of ARDS. IL-36γ neutralization in Thbs1^−/−^ mice reduced levels of proinflammatory cytokines and chemokines, improved pathogen clearance, and reduced neutrophilic activity, evaluated as lung MPO and BALF free NE activity. However, IL-36γ neutralization did not significantly reduce neutrophil recruitment to the airspaces, nor the increased production of IL-17A in the lungs. IL-17A is a master regulator of neutrophil chemotaxis (51). In a murine bacterial pneumonia model, lung IL-17 contributed to neutrophil recruitment through the induction of downstream chemokines different from CXCL1 and CXCL2, such as CXCL5 (52). Therefore, the robust production of IL-17A during P. aeruginosa infection in Thbs1^−/−^ mice treated with anti-IL-36γ may explain why IL-36γ neutralization did not reduce the increased neutrophil numbers found in the airspaces and also suggest the existence of an alternative inflammatory pathway involved in neutrophil recruitment that should be further studied.

Collectively, these data support the idea that IL-36γ neutralization could be exploited as an adjunct therapy against dysregulated inflammation observed in acute and chronic PA infections. As targeting the IL-36 signaling pathway is a viable strategy to block excessive inflammation of pustular psoriasis and other autoinflammatory disorders (53), a neutralizing antibody against IL-36R (ANB019) is currently in phase 2 clinical trial (ClinicalTrials.gov registration no. NCT03633396). One possible therapeutic application is in a subset of patients with runaway inflammation as a sequela of P. aeruginosa infection-induced tissue injury or conditions with protease/antiprotease imbalance such as in cystic fibrosis where excessive inflammation is a key feature. A better understanding of how extracellular processing of IL-36γ is regulated by the host could provide a working framework in the design of new therapeutic strategies targeting pathogenic inflammation in the lungs.

## MATERIALS AND METHODS

### Mice.

C57BL/6J (WT, stock no. 000664) mice and B6.129S2-Thbs1tm1Hyn/J (Thbs1^−/−^, stock no. 006141) mice were originally obtained from Jackson Laboratories (Bar Harbor, ME) and maintained in the animal facility of University of Pittsburgh as previously described (27, 28). Thbs1^−/−^ mice were further backcrossed an additional 5 generations before experiments. Thbs1^−/−^ and WT mice were cohoused in the same vivarium and fed the same chow for at least 4 weeks prior to *in vivo* experiments as previously described (27). All experimental protocols were reviewed and approved by the Institutional Animal Care and Use Committee (IACUC) at the University of Pittsburgh.

### Pseudomonas aeruginosa inoculation.

Pseudomonas aeruginosa strain PA14 was grown in Luria-Bertani (LB) broth to an optical density (OD) of 0.5 (1 × 10^9^ to 5 × 10^9^ CFU/ml). Then, 100 μl was resuspended in 10 ml of 1× sterile phosphate-buffered saline (PBS) reaching a final concentration of 1 × 10^7^ to 5 × 10^7^ CFU/ml. Sex matched 8- to 12-week-old WT and Thbs-1^−/−^ mice were briefly anesthetized with isoflurane in an anesthesia chamber and intratracheally (i.t.) inoculated with 1 × 10^6^ to 5 × 10^6^ CFU of PA14 in 100 μl as previously described (27). Mice were euthanatized after 5 h or 1 day.

### Bronchoalveolar lavage fluid collection.

Necropsy was performed as previously described (27–29). Briefly, the trachea was cannulated using an 18-gauge catheter, and the left lung was ligated at the hilum. Bronchoalveolar lavage fluid (BALF) was subsequently obtained from WT and Thbs1^−/−^ mice by instilling 600 μl of sterile 1× PBS-EDTA (0.6 mM), followed by 3 subsequent lavages of 500 μl (final volume of 2.1 ml) into the right lung. BALF cells were pelleted by centrifugation at 1,800 rpm for 15 min for flow cytometry, and the cell-free supernatant was collected to measure total protein content (Pierce BCA protein assay kit; Thermo Fisher), free NE, Cat S, and LasB activity.

### *Ex vivo* flow cytometry.

BALF recovered from uninfected and infected WT and Thbs1^−/−^ mice was centrifuged at 1,800 rpm for 15 min, and the pellet was incubated with 1× ammonium-chloride-potassium (ACK) buffer for 5 min at room temperature (RT) to lyse red blood cells. Cells were washed twice with 1× PBS and stained for viability (Live/Dead fixable aqua dead cell stain kit; Thermo Fisher) for 30 min at room temperature in the dark. Then, cells were washed twice with 1× PBS and resuspended in the antibody mix in PBS-newborn calf serum (NCS) 2%. The following antibodies were included in staining mix CD45-AF700 (clone 30-F11, catalog no. 560510; BD), CD11b-PE (clone M1/70, catalog no. 553311; BD), CD11c-PE-cy7 (clone HL3, catalog no. 558079; BD), CD64-BV650 (clone X54-5/7.1, catalog no. 740622; BD), CD24-BUV395 (clone M1/69, catalog no. 744471; BD), MHCII-Percp-cy5.5 (clone M5/114.15.2, catalog no. 562363; BD), Ly6C-FITC (clone AL-21, catalog no. 553104; BD), Ly6G-APC (clone 1A8, catalog no. 560599; BD), SiglecF-APC-cy7 (clone E50-2440, catalog no. 565527; BD). Samples were analyzed using a BD LSR Fortessa flow cytometer located in the unified flow core at the University of Pittsburgh. Cells were counted (cell/ml) by the addition of CountBright absolute counting beads (Thermo Fisher) immediately before samples were analyzed. Unbiased Barnes-Hut modification of *t*-distributed stochastic neighbor embedding (bh-SNE) analyses were done through the Cyt algorithm run in MatLab (http://www.c2b2.columbia.edu/danapeerlab/html/cyt-download.html) on viable CD45^+^ cells. Cell types were identified following the expression pattern of each marker used (see Fig. S1 and Table S1 in the supplemental material). In parallel, traditional gating analysis were done using FlowJo v10.6.2 Mac (Beckton Dickinson) (see Fig. S2 in the supplemental material).

10.1128/mBio.03336-20.2 FIG S2 Gating strategy for immune cell identification. Viable cells were identified with Live/Dead fixable aqua dead cell stain kit (Thermo Fisher). Leukocytes were gated based on their expression of CD45 (CD45-AF700, 30F11). The following antibodies were used: CD45-AF700 (30-F11), CD11b-PE (M1/70), CD11c-PE-cy7 (HL3), CD64-BV650 (X54-5/7.1), CD24-BUV395 (M1/69), MHCII-Percp-cy5.5 (M5/114.15.2), Ly6C-FITC (AL-21), Ly6G-APC (1A8), and SiglecF-APC-cy7 (E50-2440). Cells/ml were calculated using CountBright absolute counting beads (Life Technologies). A detailed summary of the expression pattern of surface markers used to identify each cell type can be found in Table S1 in the supplemental material. Download FIG S2, PDF file, 0.2 MB. Copyright © 2021 Peñaloza et al. 2021 Peñaloza et al. https://creativecommons.org/licenses/by/4.0/ This content is distributed under the terms of the Creative Commons Attribution 4.0 International license.

### Cytokine quantification.

Left lungs from WT and Thbs1^−/−^ mice were collected, homogenized, and resuspended in cytokine buffer (0.5% Triton X-100, 150 mM NaCl, 15 mM Tris, 1 mM CaCl_2_, and 1 mM MgCl_2_ [pH 7.40]) for 30 min at 4°C. After 30 min, samples were centrifuged at 10,000 × *g* for 20 min at room temperature (RT), and supernatants were stored at −80°C until used. Levels of G-CSF, GM-CSF, CXCL-1, CXCL-2, IL-1β, IL-6, and IL-17A (pg/ml) were measured by enzyme-limited immunosorbent assay (ELISA) using DuoSet ELISA kits (R&D) according to the manufacturer’s instructions.

### Myeloperoxidase activity.

Myeloperoxidase (MPO) activity was evaluated in the left lung tissue homogenate as previously described (27, 29). Briefly, tissue homogenates were sonicated in heated 1× hexadecyltrimethylammonium bromide (HTAB) buffer. Samples were then centrifuged, and supernatants were incubated for 1 min with *o*-dianisidine dihydrochloride and 30% hydrogen peroxide in a 96-well plate. Absorbance was read at 450 nm after 1 and 10 min. Lung MPO activity was calculated as follows: (absorbance at 10 min – absorbance at 1 min)/0.0113.

### *In vivo* LasB, neutrophil elastase, and cathepsin S proteolytic activity.

LasB activity was evaluated in BALF samples from WT and Thbs1^−/−^ by incubating BALF with 25 nM Tris, 150 nM NaCl, 10 mM CaCl_2_, and the LasB substrate (2-aminobenzoyl-l-alanyl-glycyl-l-leucyl-l-alanyl-*para*-nitro-benzyl-amide, also described as a substrate for thermolysin and neutral endopeptidase 2411 [NEP]; Peptides International) in an opaque 96-well plate (27). Then, fluorescence was measured at an excitation of 340 nm and emission of 415 nm at 0 h and after 24 h, LasB activity was calculated as follows: [RFU_340/415 (24 h)_ − RFU_340/415 (0 h)_], where RFU indicates relative fluorescence units. Purified LasB was used as a positive internal control. NE activity from BALF samples of WT and Thbs1^−/−^ mice was measured as previously described (27). Briefly, BALF samples were incubated in a clear 96-well plate with the specific NE substrate *N*-methoxysuccinyl-Ala-Ala-Pro-Val *p*-nitroanilide (15 mM) in the presence of Tris 1 M and NaCl 5 M for 24h. Absorbance was read at 410 nm at 0 h and after 24 h, and NE activity was calculated as follows: (OD_410 24 h_ − OD_410 0 h_). Purified NE was used as the positive control. CatS activity from BALF samples was measured as previously described (54). Briefly, BALF was incubated for 1 h with sodium acetate buffer supplemented with dithiothreitol (DTT) 4 mM at 37°C in a dark 96-well plate. Then, fluorogenic CatS specific substrate [Mca-GRWPPMG∼LPWEK(Dnp)-*D*-R-NH2; Millipore Sigma] was added and fluorescence was measured at an excitation of 340 nm and an emission of 405 nm at 0 h and after 24 h. CatS activity was calculated as follows: [RFU_340/405 (24 h)_ − RFU_340/405 (0 h)_]. Purified CatS was used as the positive control.

### Lung gene expression.

TRIzol RNA-based isolation was performed on snap-frozen lung tissue from WT and Thbs1^−/−^ mice. cDNA was transcribed from the isolated RNA using MultiScribe reverse transcriptase (Thermo Fisher) and *Il36a*, *Il36b*, and *Il36g* expression levels were quantified using specific TaqMan probes, *Il36a* (Mm00457645_m1, catalog no. 4331182), *Il36b* (Mm01337546_g1, catalog no. 4331182), and *Il36g* (Mm00463327_m1, catalog no. 4331182) and quantified through the threshold cycle (2^−ΔΔ^*^CT^*) method using *gadph* (Mm99999915_g1, catalog no. 4331182) as the reference gene.

### Lung IL-36γ Western blot.

Snap-frozen lung tissue (20 to 30 mg) were lysed with ice-cold Tris-based lysis buffer (Tris-HCl 50 mM, NaCl 150 mM, SDS 0.1%, Nonidet P-40 1%, and EDTA 10 mM) with complete Ultra Tablets Mini protease inhibitor (catalog no. 05892970001; Roche) and then homogenized and sonicated on ice. IL-36γ was detected using a monoclonal mouse anti-mouse IL-36γ (LS-C314326; LifeSpan Biosciences), and β-actin (catalog. no. 4970; Cell Signaling) was used as a loading control protein. The membrane was developed with chemiluminescent substrate (catalog no. 34095, Thermo Fisher), and images were captured using an Amersham 600 imager (General Electric).

### Bone marrow dendritic cell differentiation and stimulation.

Bone marrow dendritic cells (BMDCs) were obtained after culture of total bone marrow cells in the presence of GM-CSF (20 ng/ml) in RPMI medium supplemented with 10% fetal bovine serum, 2 mM glutamine, 1% penicillin-streptomycin, and 55 mM β-mercaptoethanol. Bone marrow cells were cultured at a density of 2 × 10^5^ cells/ml and medium was replaced at days 3 and 6 after culture. Adherent cells were harvested at day 9 postculture using EDTA 3 mM. BMDCs were plated at a density of 1 × 10^6^ cells/ml in 12-well plates and stimulated with 1 μg/ml of human full-length IL-36γ (2320-IL; R&D) or human cleaved IL-36γ (6835-IL; R&D) for 6 or 24 h. After each time point, cell supernatant was collected and production of IL-6 and CXCL-1 were measured by ELISA (DuoSet ELISA kits; R&D) according to the manufacturer’s instructions.

### IL-36γ *in vivo* neutralization.

Neutralizing anti-IL-36γ antibody was generated in New Zealand White rabbits that were immunized with recombinant mouse IL-36γ (30, 34). The antibody was purified and measured for titer by ELISA (30, 34). The antibody concentration administered was based on two prior studies using percent survival difference from WT mice as an endpoint, where the neutralization effect of anti-IL-36γ was shown to be equivalent to the findings observed in IL-36γ full-body knockout mice (30, 34). WT and Thbs1^−/−^ mice were intraperitoneally (i.p.) treated with 500 μl of rabbit anti-mouse IL-36γ (10 mg/ml) (30) or control rabbit IgG (SLR56; Equitech-BIO) 5 h post PA14 i.t. infection. At 1 day postinfection, lungs and BALF were collected, and bacterial burden, cytokine production, myeloid cell infiltration, and neutrophil activity were measured.

### IL-36γ *in vivo* delivery.

Recombinant cleaved human IL-36γ (2.5 μg, 6835-IL/CF, S^18^ isoform; R&D) or vehicle (PBS) were injected i.t. in WT and Thbs1^−/−^ mice in a final volume of 50 μl. At 1 day post treatment, lungs and BALF were collected, and neutrophilic recruitment, activation, and cytokine production were evaluated by cytospin (27, 29), NE/MPO activity assays, and ELISA, respectively.

### IL-36γ cleavage by P. aeruginosa and NE.

Human full-length IL-36γ (3 μg, 2320-IL-025/CF; R&D) was incubated with PBS, cell-free filtered supernatant from PA14 parent strain, or *lasB* transposon (Tn) mutant (27) for 2 h at 37°C. For LasB inhibition, the specific LasB inhibitor *N*-mercaptoacetyl-Phe-Tyr-amide (LasBI) (27) at a final concentration of 100 μM or vehicle (dimethyl sulfoxide [DMSO], 0.04%) was added. Following a 2 h of incubation, NuPAGE lithium dodecyl sulfate (LDS) sample buffer (4×) and dithiothreitol (DTT; 1 M) were added, and samples were incubated at 70°C for 10 min. Then, samples were loaded onto 12% Bis-Tris Plus gel, and MOPS [3-(*N*-morpholino)propanesulfonic acid] buffer was used as a running buffer. The gel was run at 120 V on ice. After running, the resolved gel was washed once with ultrapure water (Millipore) and then incubated overnight in QC colloidal Coomassie stain (catalog no. 1610803; Bio-Rad) at 4°C. Following overnight incubation, gel was washed with ultrapure water (Millipore) for 4 h with intermittent water changes. For N-terminal sequencing, human full-length IL-36γ was preincubated with PBS, PA14 cell-free supernatant, purified LasB (Millipore), and NE for 2h. Then samples were incubated at 70°C with LDS sample buffer (4×) and DTT (1 M) for 10 min, loaded onto 12% Bis-Tris Plus gel, and run at 120 V. Samples were shipped to the Protein Structure Core Facility (University of Nebraska Medical Center), and the first 10 amino acids of the N-terminal sequence of each product were analyzed by Edman degradation (10 cycles).

### Full-length IL-36γ and truncation models.

Using the protein amino acid sequence as input, RaptorX (deep learning-powered distance-based protein folding [55–57]) generated a 3D model of full-length IL-36γ (fIL-36γ) whose body (starting at S^18^) was a close match to the crystal structure of IL-36γ (amino acid residues 1 to 17 unresolved; PDB ID 4IZE). In PyMol Molecular Graphics System v1.3 (Schrodinger, LLC), amino acid residues were sequentially deleted from the predicted full-length IL-36γ structure to create the collection of truncation models.

### IL-36γ/IL-36R complex docking.

The program ClusPro 2.0 predicted how full-length IL-36γ and a collection of N-terminal truncation models bound to a model of the IL-36R complex. Briefly, the algorithm rotates each IL36γ model 70,000 times. and for each rotation, a series of *xyz* translations (∼10^9^ positions sampled) seats the cytokine model onto the IL-36R complex fixed on a grid. The top 1,000 best scores from this rotation/translation procedure (in the top 10^6^ of all positions tested) go forward. Cluster center identification involves using the criterion that a cluster center must include the greatest number of “neighbors” within a 9-Å C-α root mean square deviation (RMSD) radius. After this, the program takes out the previously identified neighbors before resuming the search for a second cluster center. The iterative application of the selection criterion pulls out additional cluster centers (30 total). To avoid introducing bias by assuming we knew about what forces dominate in our binding, we used the ClusPro balanced coefficients (58–60). In the evaluation of the docking results, we considered scores and cluster size, and visually inspected the docking solutions in PyMol Molecular Graphics System v1.3 (Schrodinger, LLC) to gauge relative packing efficiency. Finally, because we lacked experimental information about what amino acids should lie next to interface residues or remain solvent accessible after binding, we did not impose any restraints or constraints (attraction and repulsion) *per se* on our docking jobs.

### Statistical analyses.

For single comparisons, normal distribution between groups was evaluated using a Shapiro-Wilk test. Mann-Whitney U test analysis was used to analyze samples without normal distribution, and parametric *t* test analysis was used to analyze samples with normal distribution. Cytokine production and lung P. aeruginosa burden in WT and Thbs1^−/−^ mice were compared using single comparisons at individual time points (5 h and 1 day). Statistical significance was assigned for a *P* value of <0.05. Two-way analysis of variance (ANOVA), followed by a multiple-comparison test, was performed to analyze cellular infiltration, lung MPO activity, total BALF protein content, NE, LasB, and CatS activity in the BALF on WT and Thbs1^−/−^ mice infected with P. aeruginosa and lung cytokine production of WT and Thbs1^−/−^ mice after cIL-36γ treatment. Statistical significance was assigned for a *P* value of <0.05. One-way ANOVA test followed by a multiple-comparison test was performed to analyze the effect of IL-36γ neutralization in WT and Thbs1^−/−^ mice at 1 dpi. Statistical significance was assigned for a *P* value of <0.05. All comparisons were performed using the Prism software v8.4.3 for Macintosh (GraphPad Software).
