# Supplementary figures and images for: Thrombospondin-1 Restricts Interleukin-36γ-Mediated Neutrophilic Inflammation during Pseudomonas aeruginosa Pulmonary Infection
Source: mBio. 2021 Apr 6;12(2):e03336-20. doi: 10.1128/mBio.03336-20 (PMC8092289; doi:10.1128/mBio.03336-20)

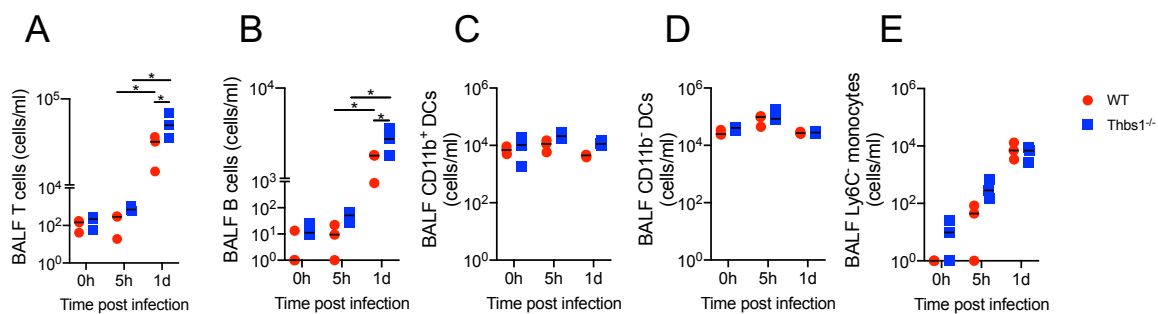

Supplement: FIG S3 [file mBio.03336-20-sf003.pdf]

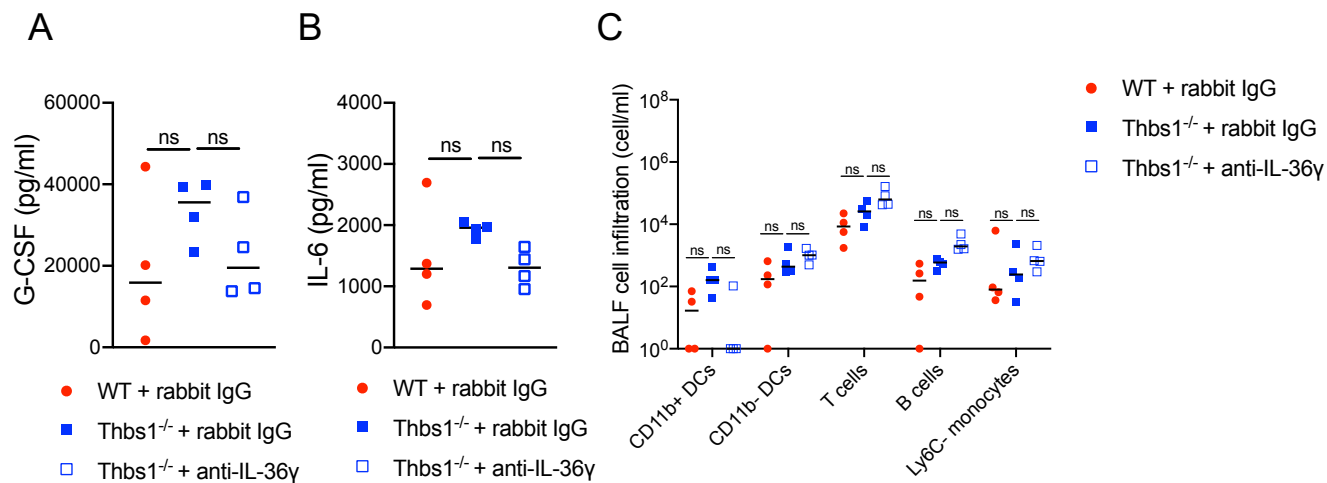

Supplement: FIG S4 [file mBio.03336-20-sf004.pdf]

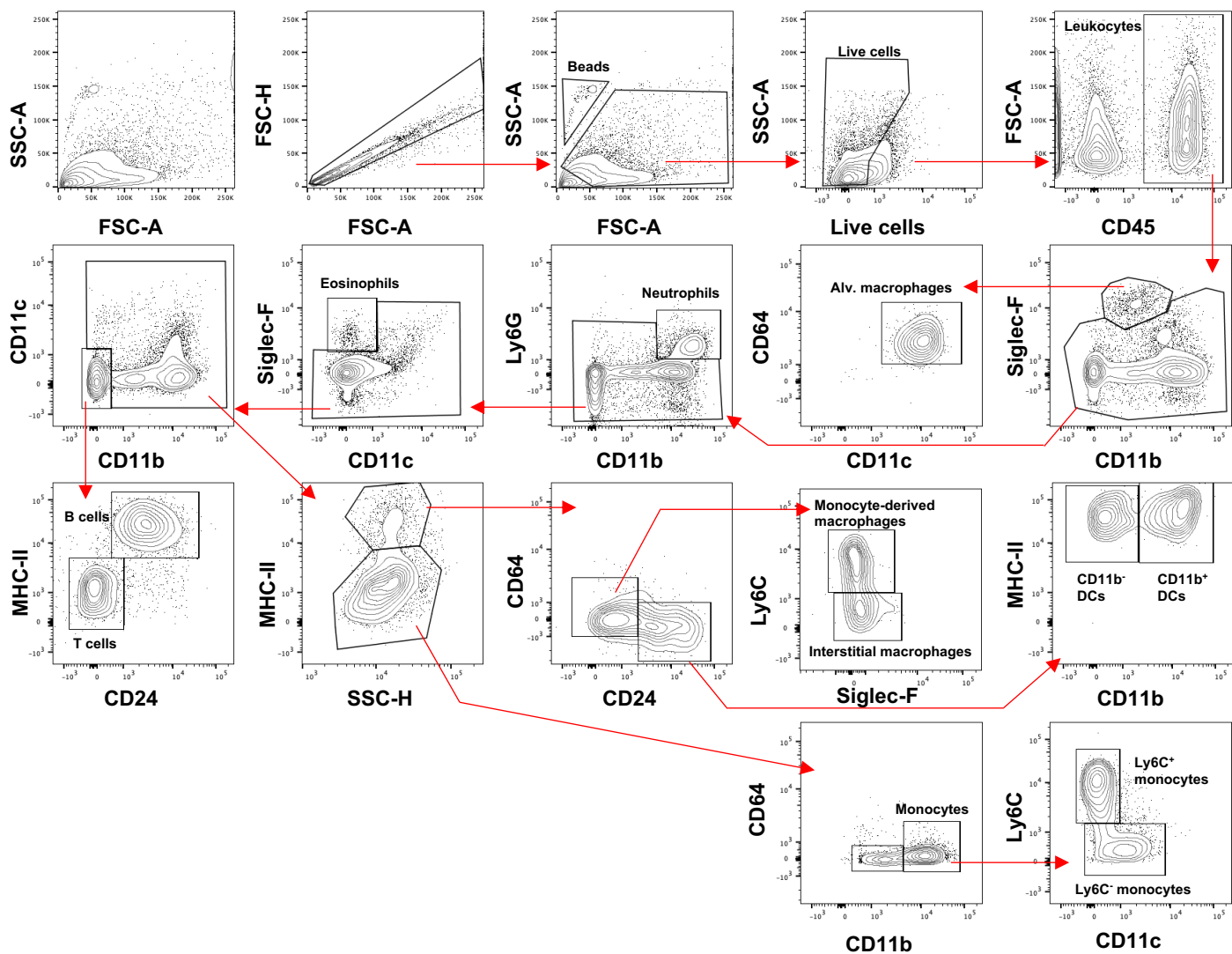

Supplement: FIG S2 [file mBio.03336-20-sf002.pdf]
