# Supplementary material for: Thrombospondin-1 Restricts Interleukin-36γ-Mediated Neutrophilic Inflammation during Pseudomonas aeruginosa Pulmonary Infection
Source: mBio. 2021 Apr 6;12(2):e03336-20. doi: 10.1128/mBio.03336-20 (PMC8092289; doi:10.1128/mBio.03336-20)
Supplement: TABLE S1 [file mBio.03336-20-st001.pdf]

Cell type	CD11b	CD11c	CD24	CD64	Ly6C	MHCII	SiglecF	Ly6G
Eosinophils	+	-	+	-	-	-	+	-
Neutrophils	+	-	+	+/-	+/-	-	-	+
Alv. Macrophages	-	+	-	+	-	+/-	+	-
Ly6C+ monocytes	+	-	-	+/-	+	-	-	-
Ly6C- monocytes	+	+/-	-	+/-	-	-	-	-
CD11b+ DCs	+	+	+	-	+/-	+	-	-
CD11b- DCs	-	+	+/-	-	+/-	+/-	-	-
T cells	-	-	-	-	-	-	-	-
B cells	-	-	+	-	-	+	-	-
Monocyte-derived macrophages	+/-	-	-	-	+	-	-	-

“+”: Positive; “-”: Negative; “+/-”: Medium expression.
