# Supplementary material for: Thrombospondin-1 Restricts Interleukin-36γ-Mediated Neutrophilic Inflammation during Pseudomonas aeruginosa Pulmonary Infection
Source: mBio. 2021 Apr 6;12(2):e03336-20. doi: 10.1128/mBio.03336-20 (PMC8092289; doi:10.1128/mBio.03336-20)

A

Bh-SNE analysis BALF 0h, 5h, 1dpi WT and $Thbs1^{-/-}$ mice

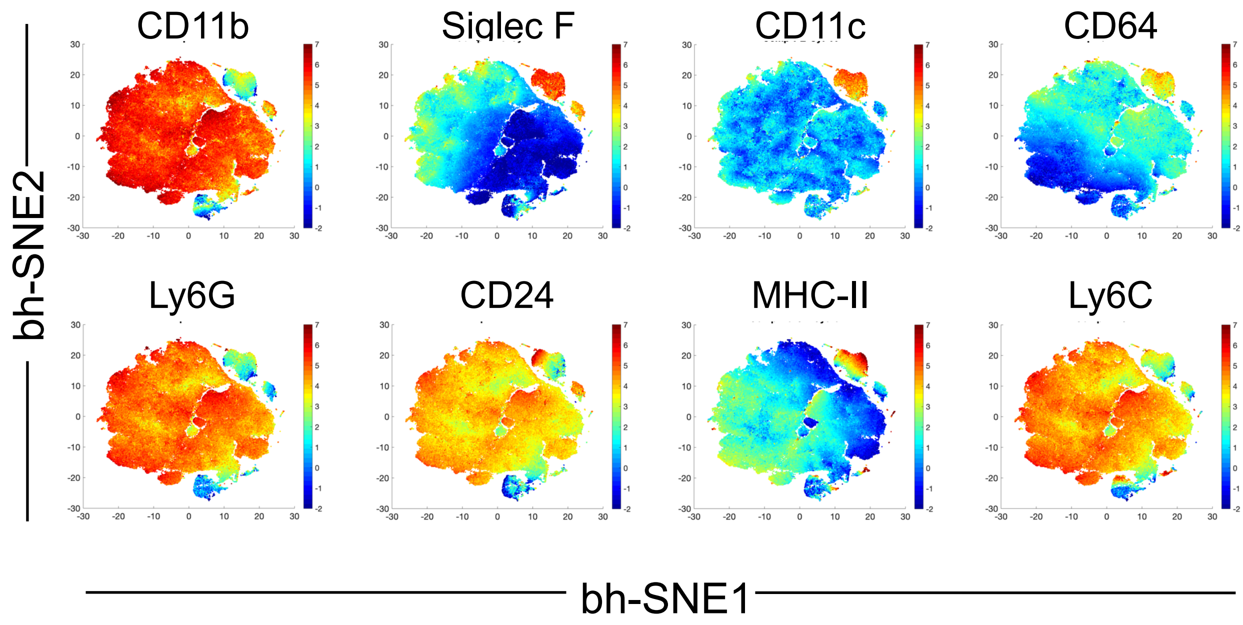

B

Bh-SNE analysis BALF 1dpi WT and $Thbs1^{-/-}$ mice anti IL-36γ

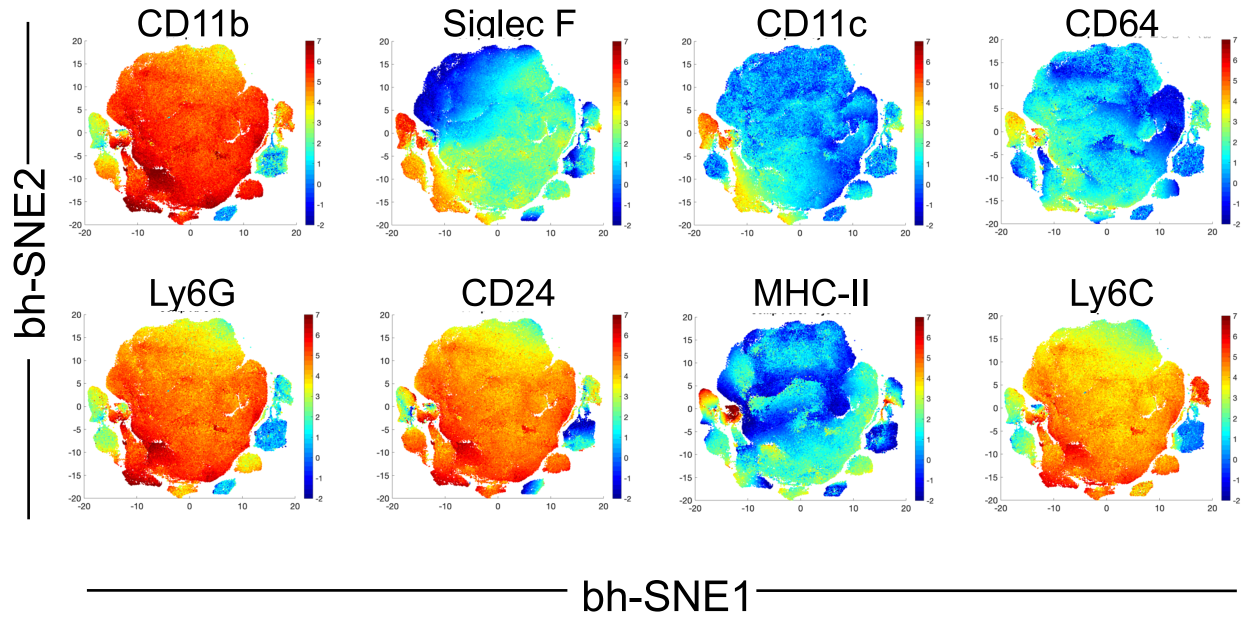

Supplement: FIG S1 [file mBio.03336-20-sf001.pdf]
